# The Neighbourhood Built Environment and Trajectories of Depression Symptom Episodes in Adults: A Latent Class Growth Analysis

**DOI:** 10.1371/journal.pone.0133603

**Published:** 2015-07-24

**Authors:** Genevieve Gariepy, Brett D. Thombs, Yan Kestens, Jay S. Kaufman, Alexandra Blair, Norbert Schmitz

**Affiliations:** 1 Institute for Health and Social Policy, McGill University, Montreal, Quebec, Canada; 2 Department of Psychiatry, McGill University, Montreal, Quebec, Canada; 3 Department of Social and Preventative Medicine, University of Montreal, Montreal, Quebec, Canada; 4 Department of Epidemiology, Biostatistics and Occupational Health, McGill University, Montreal, Quebec, Canada; Arizona State University, UNITED STATES

## Abstract

**Aim:**

To investigate the effect of the neighbourhood built environment on trajectories of depression symptom episodes in adults from the general Canadian population.

**Research Design and Methods:**

We used 10 years of data collection (2000/01-2010/11) from the Canadian National Population Health Study (n = 7114). Episodes of depression symptoms were identified using the Composite International Diagnostic Interview Short-Form. We assessed the presence of local parks, healthy food stores, fast food restaurants, health services and cultural services using geospatial data. We used latent class growth modelling to identify different trajectories of depression symptom episodes in the sample and tested for the effect of neighbourhood variables on the trajectories over time.

**Results:**

We uncovered three distinct trajectories of depression symptom episodes: low prevalence (76.2% of the sample), moderate prevalence (19.2%) and high prevalence of depression symptom episodes (2.8%). The presence of any neighbourhood service (healthy food store, fast-food restaurant, health service, except for cultural service) was significantly associated with a lower probability of a depression symptom episode for those following a trajectory of low prevalence of depression symptom episodes. The presence of a local park was also a significant protective factor in trajectory groups with both low and moderate prevalence of depression symptom episodes. Neighbourhood characteristics did not significantly affect the trajectory of high prevalence of depression symptom episodes.

**Conclusions:**

For individuals following a trajectory of low and moderate prevalence of depression symptom episodes, the neighbourhood built environment was associated with a shift in the trajectory of depression symptom episodes. Future intervention studies are recommended to make policy recommendations.

## Introduction

Depression is a prevalent public health concern and the leading cause of disability in the world [1]. It is estimated that about 5% of the population in Canada and other Western countries currently have major depression [2, 3]. Research has identified several individual-level risk factors of depression, but there has been growing interest in the larger neighbourhood-level factors that contribute to depression. Several studies have shown neighbourhood characteristics to be significantly associated with depression [4, 5]; however, evidence has been mainly cross-sectional. In studies using longitudinal data, findings have been less consistent, with about half reporting a significant association [4–6]. Variation in depression and neighbourhood over time might explain some of the mixed findings. Longitudinal studies have typically measured neighbourhood characteristics at baseline, and modelled depression event at only one time point. Neighbourhoods may change over time and people may change which neighbourhood they live in. Depression also follows a dynamic process characterized by different trajectories [7]. Research that takes into account the changing nature of neighbourhoods and depression over time has therefore been recommended as the next step for clarifying the role of neighbourhood in depression [4, 7, 8]. This line of research could not only provide insight into the risk factors of depression, but also into factors that contribute to certain long-term depression patterns.

Among the different characteristics of neighbourhoods, studies have found some evidence that the built environment—the physical structures and infrastructures in the local environment, such as parks, buildings, and stores—is linked to mental health [9, 10]. Neighborhood features such as local parks could offer respite from stress and a place to foster social connections [11]. The proximity to certain local businesses and services could also be important, such as nearby health services which could facilitate access to mental healthcare. Nonetheless, research on the effect of the built environment specifically in the context of depression has been limited and evidence has so far been cross-sectional. Kubzansky et al. reported no significant association between depressive symptoms and neighbourhood services promoting social engagement, services providing care, and undesirable amenities, in an elderly sample [12]. Stockdale et al. found no association between depression and number of alcohol outlet and number of alcohol, drug, and mental-health facilities in a sample of adults [13]. Longitudinal evidence is missing and the effect of other aspects of the built environment (such as parks and healthy food stores) on depression over time has not been examined.

Using 10 years of data collection from a large national population health survey, the objective of this study was to identify trajectories of depression symptom episodes in adults and assess whether aspects of the built environment affect the trajectories over time. We used latent class growth modelling to identify different trajectories of depression symptom episodes. We hypothesized that characteristics of the built environment would impact depression trajectories.

## Methods

### Study population

We used 10 years of data from the National Population Health Survey (NPHS) [14]. The NPHS is a large population-based health survey of individuals across Canada (baseline n = 17,276), with 9 cycles of data collected from 1994/95 to 2010/11. Participants were selected using a stratified cluster sampling strategy. Follow-up interviews were conducted every 2 years. Details on the NPHS can be found elsewhere [14]. For this study, we used data from 2000/2001-2010/2011, corresponding to 6 survey cycles (cycles 4 to 9), because data on the neighbourhood built environment were available from 2000/2001 onwards. Response rates were 85%, 81%, 78%, 77%, 71% and 70%, for each of the survey cycles, respectively (S1 Table). To insure comparability with other studies, we included adults who were between the ages of 18 and 80 at baseline (n = 13,618). Our final analytic model was restricted to those without missing data on baseline covariates (n = 7114).

### Ethics Statement

The study was approved by the Research Ethics Committee of the Douglas Mental Health University Institute. Written informed consent was obtained from all participants.

### Measures

#### Episode of depression symptoms

The outcome of interest was past-year episode of major depression symptoms assessed using the validated Composite International Diagnostic Interview Short-Form for Major Depression (CIDI-SFMD)[15]. Participants were asked whether they experienced depressed mood or loss of interest for at least 2 consecutive weeks in the past year. Those who endorse one of these two key depression symptoms were then asked about eight other depressive symptoms. Criteria for an episode of major depression symptoms require the endorsement of at least five depressive symptoms, including depressed mood or loss of interest, as described in the Diagnostic and Statistical Manual of Mental Disorders (DSM-IV)[16]. The CIDI-SFMD is a brief screening tool used to identify a high probability of a major depression episode, but is not a clinical interview that yields a full diagnosis of major depression. Previous studies have shown that 75% to 90% of subjects identified has having an episode of major depression symptoms on the CIDI-SFMD had major depression according to the full CIDI [15, 17].

#### Neighbourhood characteristics

Neighbourhoods were defined using a person-centered approach. We chose a 500m radius buffer around the center of the postal code of each participant based on previous work [18] and measured the neighbourhood characteristics within the zone. The postal code is a six-character alphanumeric code that forms part of the postal address in Canada and denotes the local postal delivery unit. It may indicate a specific city block, a single building, or a large volume mail receiver in urban areas, and a rural community in rural areas [19]. The Canadian postal code has been shown to be a good proxy for the full home address [20]. Geospatial Canadian data from 2002, 2006 and 2010 were used to estimate density of businesses and services, as well as parks and recreational facilities [21]. Business and service data included the number of health services, healthy food stores, fast-food restaurants and cultural services (see S1 Text for details). Parks and recreation data were modelled as the proportion of an area used for parks, sports tracks, or swimming pools. We dichotomized density of businesses and parks and recreational facilities as absent (0%) versus present (>0%) in the neighbourhood because data were extremely skewed. Neighbourhood data that were closest in time to each survey cycle were used to approximate the neighbourhood characteristics of that survey. Data on neighbourhood businesses were available for 99% of the sample and data on parks and recreation were available for 72% of the sample.

#### Sociodemographic and health variables

Based on our literature review, we investigated variables known to be important to both neighbourhood selection and depression symptoms. We used these variables to characterize and compare the different trajectories of depression episodes. We included baseline information on sex, age (<42 years; ≥ 42 years, the median age), marital status (partnered: married, common law; not partnered: single, widowed, divorced, separated), education (less than high school; high school graduation or higher), family income adequacy (based on the number of people in the household and on Statistics Canada's low-income cut-offs [22]; low vs middle/ high), family history of depression (yes/no), chronic condition (none; any of the following: asthma, chronic bronchitis, heart disease, arthritis, cancer, back pain, high blood pressure, migraines, stomach/intestinal ulcers, bowel disorder, thyroid condition) and childhood life events (none; any of the following: two weeks or more in hospital; parents divorced; parents unemployed for a long time; traumatised for years; sent away from home; parents abused alcohol or drugs; was ever physically abused).

### Statistical analysis

We identified trajectories of prevalence of depression symptom episodes in the sample from latent class growth modelling (LCGM) using a logistic model [23]. LCGM is a semi‐parametric technique used to uncover distinct groups of individuals who follow a similar pattern of change over time. In a first step, we fitted a one-class solution to the data, equivalent to the null hypothesis that all participants in the NPHS follow the same trajectory of probability of depression symptom episodes. We then added more classes until the number of groupings reached a good fit with the data. The final number of classes was selected based on model fit indices (Bayesian Information Criterion (BIC), Akaike information criterion (AIC)), interpretability of the model, and meaningfulness of each class. The shape of the trajectories in the final model was selected based on the significance of the polynomial components of each class [23].

In a second step, we included into the model individual-level sociodemographic and health characteristics that affected the probability of group membership and directly tested their association with each trajectory group. This approach takes into account the uncertainty of group assignment based on probability [24].

In a third step, we examined how changes in neighbourhood characteristics over time altered the trajectory of depression symptom episodes within a group by introducing time-varying neighbourhood characteristics in the model. We checked if the effects of the time-varying neighbourhood factors were also time-dependent by including an interaction term between the neighbourhood variable and log-time. We found no evidence of a time-dependent effect. The coefficients of the time-varying neighbourhood variables are interpreted the same way as an ordinary logistic regression, but within each trajectory group, and represent the deviation in the long-term probability of depression of members of that group. Neighbourhood variables were entered one at a time.

#### Additional analyses

We conducted additional analyses to check the consistency of our findings. We classified participants in the trajectory group for which they had the highest probability of belonging and used a multinomial logistic regression with the depression trajectory groups as outcomes to investigate the association between neighbourhood factors in 2002, 2006 and 2010 and trajectory membership. The association between changes in neighbourhood characteristics and depression trajectories among movers might differ from non-movers. We therefore conducted a sensitivity analysis restricting the sample to non-movers during the 10-year follow-up (n = 3553, identified from changes in postal code). We conducted a restricted analysis to non-rural dwellers (n = 5501, determined from postal codes) because rural dwellers may have different life situations than urban dwellers (e.g., different access to healthcare) and data from rural areas are more difficult to accurately obtain. Results were similar to the full sample. We also investigated the difference in fit for our final model (using BIC and AIC) between the LCGM and a regular growth mixture model that allowed for random effects within class, in MPlus (version 7.0, Los Angeles, CA). We found no marked difference in fit between these models (LCGM: BIC: 16788.041, AIC: 16729.632; growth mixture model with random effects: BIC: 16809.340, AIC: 16729.028).

There was some difference in sociodemographic characteristics between those with and without missing data. Those without missing data were more likely to be women (54.1% vs. 47.1%, p<0.001), younger (42.2% vs. 49.6% older than 42 years old, p<0.001), with a partner (71.1% vs 63.0%, p<0.001), to have secondary education or more (80.7% vs 69.5%, p<0.00) and to have a family history of depression (29.8% vs 26.1%, p = 0.04), compared to those with missing data. Missing data were mainly attributable to missing information on family history of depression and income adequacy. To test the impact of missing values, we reran analyses adjusting only for age, sex and education status, and analyses using multiple imputations.

LCGM was performed in STATA (version 12.1, College Station, TX) using the traj procedure [24]. The traj procedure assumes that missing data on the dependent variable (i.e., depression symptom episode) are missing at random and therefore includes subjects with some missing data on the dependent variable in analysis, but it removes subjects with missing data on covariates. All analyses were conducted using study weights provided by Statistics Canada to adjust for non-response and lost to follow-up, and to account for the complex survey design of the survey [14].

## Results

Participants in the sample were 43 years old on average at baseline (2001/01) and the majority were partnered, working and had middle to high income adequacy (Table 1). Prevalence of depression symptom episodes at each survey cycle was 4.5%, 4.9%, 4.7%, 4.2%, 4.0% and 3.9% in 2000/01, 2002/03, 2004/05, 2006/07, 2008/09 and 2010/11, respectively.

**Table 1:** Association between baseline characteristics and trajectory class of depression symptom episodes. All estimates were weighted using Statistics Canada survey weights.

	All participants	Trajectory 1	Trajectory 2	Trajectory 3
	n = 7,114	Low prevalence of depression symptom episodes	Moderate prevalence of depression symptom episodes	High prevalence of depression symptom episodes
Variables	% (n)	Estimate (95% CI)	Estimate (95% CI)	Estimate (95% CI)
		(reference)		
**Sex**				
Male (reference)	45.9% (3269)			
Female	54.1% (3845)	1	1.01 (0.52, 1.50)	0.46 (-0.72, 1.64)
**Age**				
<42 years (reference)	57.8% (4110)			
≥42 years	42.2% (3004)	1	-0.89 (-1.28, -0.50)	-1.42 (-2.09, -0.75)
**Marital Status**				
Not partnered (reference)	71.1% (5056)			
Partnered	28.9% (2058)	1	-0.4 (-0.85, 0.05)	-1.02 (-1.71, -0.33)
**Education**				
Secondary school graduation or higher (reference)	19.3% (1371)			
Less than secondary school	80.7% (5743)	1	-0.19 (-0.72, 0.34)	-0.5 (-1.34, 0.34)
**Household income adequacy**				
Low (reference)	8.1% (579)			
Middle/high	90.2% (6535)	1	-0.37 (-1.08, 0.34)	-1.14 (-1.96, -0.32)
**Family history of depression**				
No (reference)	70.2% (4997)			
Yes	29.8% (2117)	1	0.96 (0.57, 1.35)	1.94 (1.04, 2.84)
**Childhood life events**				
None (reference)	49.9% (3549)			
1 or more	50.1% (3565)	1	0.89 (0.4, 1.38)	0.93 (0.20, 1.66)
**Chronic condition**				
None (reference)	33.8% (2405)			
1 or more	66.2% (4709)	1	0.67 (0.22, 1.12)	1.35 (0.43, 2.27)

**Fig 1:**
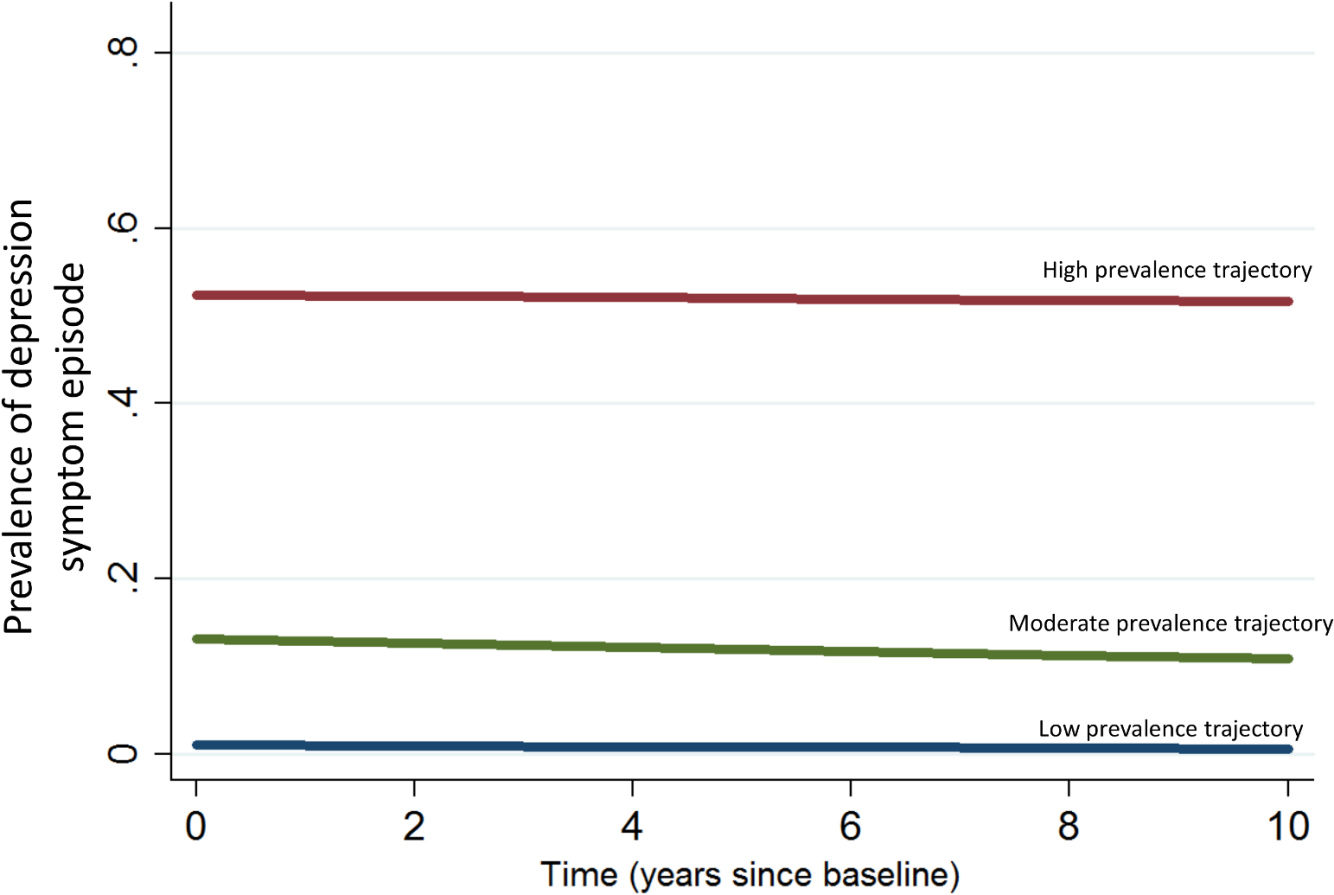
Trajectories of probability of depression symptom episodes over time in the NPHS (2000/01-2010/11). Trajectories from a 3-class latent class growth model incorporating age, sex, marital status, education, income adequacy, childhood life events, chronic condition and family history of depression. The red line represents the trajectory with high prevalence of depression symptom episodes; the green line, moderate prevalence; the blue line, low prevalence.

We selected a three-class solution for the LCGM, based on fit indices (S2 and S3 Tables), interpretability, and meaningfulness. The final model included three linear trajectories (Fig 1). Associations of sociodemographic and health variables with trajectory groups are presented in Table 1. Trajectory 1 was the largest class (76.2% of the sample; average probability of class membership: 0.88, 99% confidence interval (CI) 0.88–0.89) and represented individuals who followed a trajectory of low prevalence of depression symptom episodes during the study period. Expected prevalence of depression symptom episodes stayed consistently below 1% for every survey cycle within this group. Compared to the two other groups, members of this group were on average older and less likely to have a family history of depression, a chronic condition and to report a traumatic childhood life event. Trajectory 2 represented individuals who had a moderate prevalence of depression symptom episodes (19.2% of the sample; average probability of class membership: 0.74, 99% CI 0.73–0.75). Expected prevalence of depression symptom episodes for trajectory 2 was 20%, 19%, 24%, 15%, 21% and 17% in 2000/01, 2002/03, 2004/05, 2006/07, 2008/09 and 2010/11, respectively. The average member of this group had 1.2 episodes of depression symptoms during the study. Compared to trajectory 1, those in trajectory 2 were younger, and more likely to be a woman. Trajectory 3 grouped individuals who had a high prevalence of depression symptom episodes during the study (2.8% of the sample; average probability of class membership: 0.80, 99% CI 0.76–0.83). Expected prevalence of depression symptom episodes for trajectory 3 was 59%, 68%, 61%, 62%, 62% and 66% in 2000/01, 2002/03, 2004/05, 2006/07, 2008/09 and 2010/11, respectively. Members of this group had an average of 3.8 events of depression symptoms during the study. Compared to trajectory 1, individuals who followed trajectory 3 were younger, less likely to have a partner and more likely to have low household income adequacy. Members of this group were also likelier than any other group to report a family history of depression (Wald test comparing parameter estimate of group 3 vs group 1, p<0.001; and group 3 vs group 2, p = 0.05).

**Fig 2:**
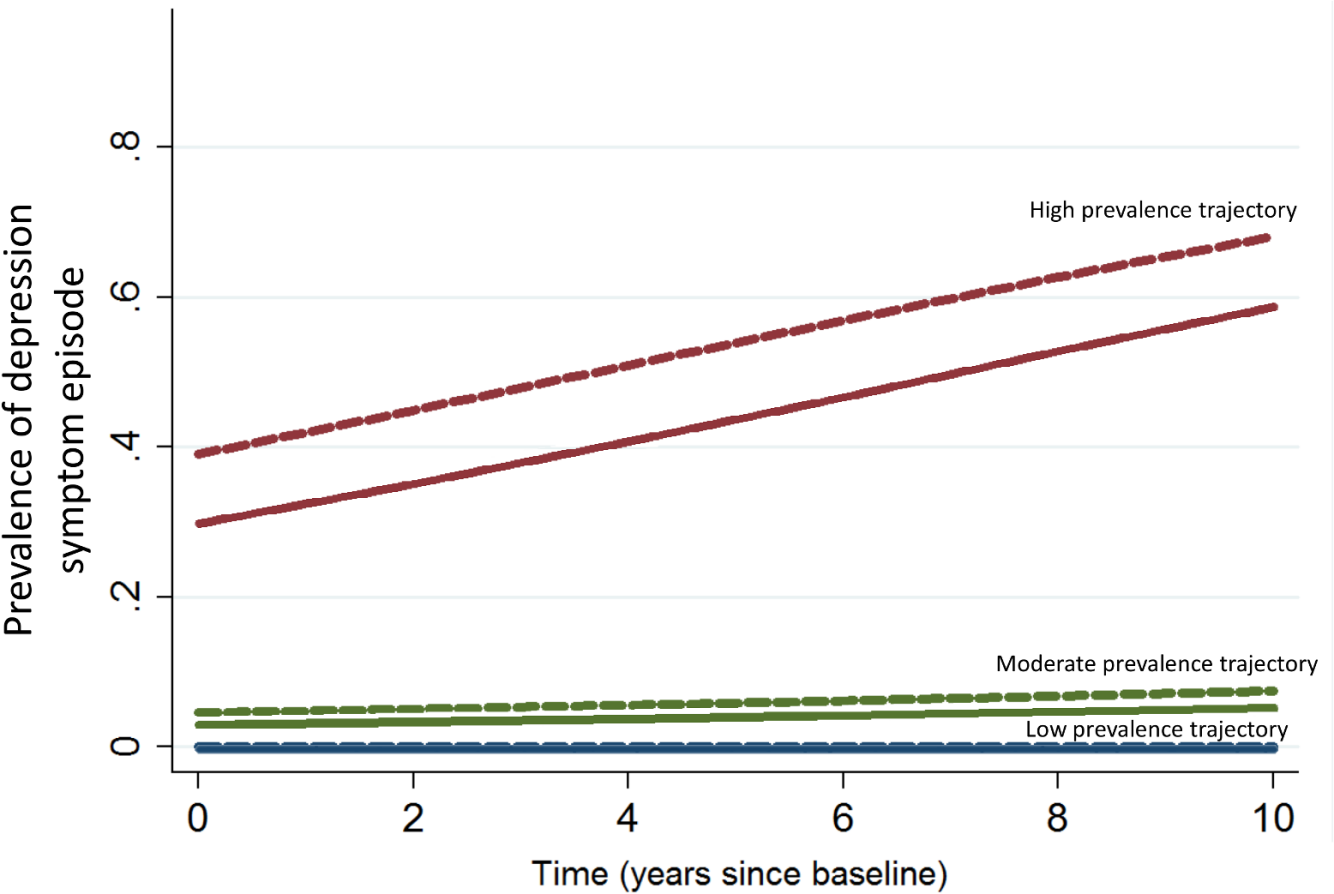
Trajectories of prevalence of depression symptom episodes over time in the NPHS (2000/01-2010/11) with and without presence of parks in the neighbourhood during the study period. Trajectories that include time-varying presence of parks in the growth model. The dashed lines represent trajectories when presence of park is set to “no park” across the study period. The solid lines represent trajectories when presence of park is set to “presence of park” across the study period.

When introducing time-varying neighbourhood characteristics into the LCGM (Table 2), the presence of any neighbourhood service (including presence of parks, healthy food stores, fast food restaurants and health services), except for cultural services, was significantly associated with a trajectory shift towards a lower probability of having a depression symptom episode in those already following a low probability trajectory of depression symptom episodes. The presence of parks was also significantly associated with a shift towards a lower probability of a depression symptom episode in those following a moderate probability trajectory. Fig 2 illustrates the probability of depression symptom episodes when local parks are modelled as absent vs. present throughout the study period. Point estimates suggest that the presence of parks was associated with a 95% lower odds of having a depression symptom episode for the group following a low probability trajectory of depression symptom episodes and a 26% lower odds for the group following moderate probability of depression symptom episodes (Table 2). None of the neighbourhood characteristics were significantly associated with the trajectory of high prevalence of depression symptom episodes.

**Table 2:** Association of time-varying neighbourhood variables with the log-odd of having an episode of depressive symptoms over the study period within each trajectory class. All models were weighted using Statistics Canada survey weights and included age, sex, marital status, education, income adequacy, childhood life events, chronic condition and family history of depression.

	Trajectory 1	Trajectory 2	Trajectory 3
	Low prevalence of depression symptom episodes	Moderate prevalence of depression symptom episodes	High prevalence of depression symptom episodes
**Neighbourhood characteristics**	Coefficient (95% CI)	Coefficient (95% CI)	Coefficient (95% CI)
**Presence of any park**	-3.1 (-3.9, -2.2)	-0.3 (-0.6, -0.01)	-0.4 (-1.4, 0.6)
**Presence of any healthcare service**	-1.9 (-3.0, -0.7)	-0.3 (-0.7, 0.1)	0.4 (-0.2, 1.0)
**Presence of any healthy food store**	-2.6 (-3.5, -1.6)	-0.2 (-0.5, 0.1)	-0.1 (-0.8, 0.6)
**Presence of any fast food restaurant**	-4.9 (-6.2, -3.7)	0.01 (-0.3, 0.3)	0.2 (-0.4, 0.8)
**Presence of any cultural service**	0.7 (-4.4, 5.8)	0.1 (-0.3, 0.4)	0.1 (-0.8, 1.0)

In additional analyses (S5 Table), we found that presence of health services and healthy food stores at baseline (in 2002) were associated with a greater probability of belonging to the trajectory with low prevalence of depression symptom episodes compared to those with a moderate prevalence of depression symptom episodes. However, living in a neighbourhood with more health services at the end of the study (in 2010) was significantly associated with having belonged to the trajectory of high prevalence of depression symptom episode during the study period. In analysis restricted to non-movers (S5 Table), direction of associations between time-varying neighbourhood characteristics and trajectories of depression symptom episodes remained largely unchanged, but statistical significance was lost. One exception was the presence of parks which remained significant for those following a moderate prevalence trajectory of depression symptom episodes. In analysis among non-rural dwellers, results were similar and in the same direction as the full sample (results omitted due to Statistics Canada disclosure rules).

In sensitivity analyses for missing data, results from the model adjusting only for age, sex and education, were largely similar as the fully adjusted model, except that the presence of parks was not significant in any of the trajectories. Using multiple imputation on the data also provided similar results as those from the complete set, but confidence intervals were wider (results not shown).

## Discussion

This study sought to examine the association between aspects of the neighbourhood built environment and trajectories of depression symptom episodes over time in a community sample of adults from the Canadian population. Analyses revealed three distinct trajectories in the sample during the 10-year follow-up, including low, moderate and high prevalence of depression symptom episodes. Others have found similar trajectories of prevalence of depression episodes in samples of adults [25, 26]. The presence of parks, healthy food stores, fast food restaurants and health services were all significantly associated with a lower probability of a depression symptom episode in those that already followed a pattern of low prevalence of depression symptom episodes. Living in an area with a local park was also significantly associated with a lower probability of a depression symptom episode in those that followed a trajectory of moderate prevalence of depression symptom episodes. None of the neighbourhood factors significantly affected the trajectory of high prevalence of depression symptoms. This study is the first to provide evidence that neighbourhood characteristics are associated with trajectories of depression symptom episodes over time in adults. These findings lend support to the notion that some aspects of neighbourhoods play a role in depression in adults [4, 5].

The presence of any neighbourhood service, including parks, healthy food stores, fast food restaurants and health services, was associated with a significant shift towards a lower probability of a depression symptom episode in those following a trajectory of no to low prevalence of depression symptoms, but not in those following other trajectories. Individuals with a low probability of a depression symptom episode might be in a better position to enjoy the mental health benefits of their neighbourhood built environment. For instance, depression is often accompanied by fatigue and loss of energy [16]. Individuals who rarely or never have depression symptoms may therefore have more energy to go to the park, to meet friends at a restaurant and to take advantage of health services. It is hypothesized that many neighbourhood structures protect mental health in part by providing opportunities for people to meet and expand their social support network [27]. However, depression affects self-esteem and the ability to connect with others [16]. Individuals who do not experience depression symptoms may therefore be more likely to benefit from opportunities to socially connect. Living in proximity to a park during the course of the study was associated with a lower odds of a depression symptom episode both in those following a pattern of low and moderate prevalence of depression symptom episodes. Local parks could offer a place to unwind from stress, to engage socially with others or in physical activities, all of which could contribute to a lower risk of depression symptoms [10, 28, 29].

The study identified a small but important subgroup of individuals who followed a pattern of high prevalence of depression symptom episodes. Similar to our results, previous studies report that about 20% of patients with major depression develop a chronic course of depression [30, 31]. Individuals who have persistent and recurrent major depression symptoms are a particularly vulnerable group to a host of poor health outcomes, functional problems and lost life opportunities [32, 33]. We found no evidence in this study that neighbourhood factors could change the trajectory of high persistent depression symptoms. In sensitivity analysis, those who followed this a trajectory were more likely to live near a health service at the end of the study, perhaps because of greater healthcare needs following years of chronic depression symptoms. One explanation could be the small sample size of this trajectory group which could limit the power to statistically detect this effect. Alternatively, depressive symptoms in individual with recurrent episodes of depression symptoms may in fact be unaffected by the built environment. These findings are in line with previous research that suggests that the main predictors of chronic depression are personal and family factors, such as younger age of depression onset, longer duration of depressive symptoms and family history of mood disorder. [30]

This study is strengthened by the use of a large community sample of Canadian adults, longitudinal data covering 10 years of data collection with multiple assessment points, and the careful consideration of the time-varying nature of neighbourhood characteristics and depression. We used objective measures of the neighbourhood built environment and a person-centered definition of neighbourhoods. This approach is thought to capture the neighbourhood environment more accurately than administrative geographic units (e.g., census tracts), but may differ from what people consider their neighbourhood. Future research testing self-defined neighbourhoods might be useful. We uncovered depression trajectories using latent class growth modelling.

Several limitations should nonetheless be noted. The latent class growth model identified three trajectory groups of major depression in the sample, but these groups are not fixed and members of these groups are not expected to follow the same trajectory permanently. Different trajectories may be identified in different populations and within the same population at different time points. Research replicating findings in other populations is recommended. Although the CIDI-SFMD is a validated instrument to assess major depression symptoms, it is a screening tool and not a clinical interview. The study examined a range of characteristics of the neighbourhood built environment, but other aspects of the neighbourhood could also be important. Although we investigated several baseline individual-level variables in the association between trajectories of depression symptom episodes and neighbourhoods, some of these variables may vary with time, and may further affect and be affected by neighbourhood characteristics. Future research that incorporates methods to appropriately handle time-varying confounders is recommended to more fully account for individual-level confounding. Alternate modelling strategies might also be helpful to explore the relationship between changing neighbourhood environments and depressive symptoms, such as latent transition analysis and repeated measures latent class analysis. Residual confounding by other neighbourhood-level variables is also possible, such as neighbourhood poverty. There was no information on residential mobility between neighborhoods, or what neighborhood exposures participants experienced before the study began. Nonetheless, the study covered up to 10 years of residential history.

Characteristics of the built environment such as neighbourhood services and parks were significantly associated with shifts in the trajectories of depression symptom episodes towards a lower prevalence of depression symptom episodes in a sample of Canadian adults, particularly in those that followed a trajectory of low probability of depression symptom episodes over time. Although the latter group already enjoyed a low prevalence of depression symptom episodes, it represented the largest group (76.2% of the sample). The promotion of mental health through neighborhood services and greenspace could thus have an important impact of population-level mental health. Future intervention and impact studies are recommended to make public health recommendations.

## Supporting Information

S1 Text Details on classification of business and service data. (DOCX)

S1 Table Sample size of participants in study with information on depressive symptoms at each survey cycle of the NPHS (2000/01-2010/11). (DOCX)

S2 Table Comparison of fit statistics for 1- to 4-class solutions. BIC: Bayesian Information Criterion; AIC: Akaike information criterion. *Smaller absolute values indicate a better balance between fit and parsimony
(DOCX)

S3 Table Parameter estimates for latent class growth model of major depression using 3-class solution. Model was weighted using Statistics Canada survey weights and incorporated for age, sex, marital status, education, income adequacy, childhood life events, chronic condition and family history of depression.
(DOCX)

S4 Table Associations between neighbourhood characteristics in 2002, 2006 and 2010 and trajectory membership. Model was weighted using Statistics Canada survey weights and incorporated for age, sex, marital status, education, income adequacy, childhood life events, chronic condition and family history of depression.
(DOCX)

S5 Table Association of time-varying neighbourhood variables with the log-odd of a depression symptom episode within each trajectory class, in study participants who did not move. All models were weighted using Statistics Canada survey weights and incorporated for age, sex, marital status, education, income adequacy, childhood life events, chronic condition and family history of depression.
(DOCX)
