# Supplementary material for: The Neighbourhood Built Environment and Trajectories of Depression Symptom Episodes in Adults: A Latent Class Growth Analysis
Source: PLoS One. 2015 Jul 24;10(7):e0133603. doi: 10.1371/journal.pone.0133603 (PMC4514736; doi:10.1371/journal.pone.0133603)
Supplement: S1 Text — (DOCX) [file pone.0133603.s001.docx]

**S1 Text. Details on classification of business and service data.**

Desktop Mapping Technologies Inc. (DMTI software, Markham, Ontario, Canada, 2010) provided data on operating businesses across Canada. We developed specific algorithms using Standard Industrial Classification (SIC) codes in the DMTI databases to identify the number of healthcare services, healthy food stores, fast-food restaurants, and cultural services in neighbourhoods. Healthcare services include those covered by the Canada Health Act, which largely includes care delivered in hospitals and by physicians. Healthy food stores included stores that offer a selection of fruits and vegetables, meats, fish and/or seafood. Fast-food restaurants were restaurants that served food prepared and served quickly and often high in fat and caloric content. We identified fast food restaurants, outlets and retailers using keywords that corresponded both to fast food chain names as well as qualifying terms such as “fried”. Cultural services were establishments that contributed to the local culture, including libraries, museums and botanical gardens. Keywords were searched for anywhere in the “name” field of the DMTI database. Unequivocal keyword terms or chain names were searched regardless of SIC code to maximize coverage. Potentially equivocal keyword terms were searched only under relevant SIC codes, avoiding countless false positives. Although seemingly relevant SIC classification exist in the database (e.g., SIC for health services), some did not meet our needs (e.g., too many missing or unwanted entities), and were therefore not included in their entirety.
