# Supplementary material for: The Neighbourhood Built Environment and Trajectories of Depression Symptom Episodes in Adults: A Latent Class Growth Analysis
Source: PLoS One. 2015 Jul 24;10(7):e0133603. doi: 10.1371/journal.pone.0133603 (PMC4514736; doi:10.1371/journal.pone.0133603)
Supplement: S1 Table — (DOCX) [file pone.0133603.s002.docx]

**S1 Table. Number of participants included in study with information on depressive symptoms at each survey cycle of the NPHS (2000/01-2010/11).**

| NPHS Survey years | Sample size |
| --- | --- |
| 2000-01 | 6704 |
| 2002-03 | 6409 |
| 2004-05 | 6744 |
| 2006-07 | 5909 |
| 2008-09 | 5398 |
| 2010-11 | 5159 |
