# Supplementary material for: The Neighbourhood Built Environment and Trajectories of Depression Symptom Episodes in Adults: A Latent Class Growth Analysis
Source: PLoS One. 2015 Jul 24;10(7):e0133603. doi: 10.1371/journal.pone.0133603 (PMC4514736; doi:10.1371/journal.pone.0133603)
Supplement: S2 Table — BIC: Bayesian Information Criterion; AIC: Akaike information criterion. *Smaller absolute values indicate a better balance between fit and parsimony (DOCX) [file pone.0133603.s003.docx]

**S2 Table. Comparison of fit statistics for 1- to 4-class solutions**

| Number of classes | Polynomial order of coefficients for best model | BIC* | AIC* |
| --- | --- | --- | --- |
| 1 | 2 | -9208.81 | -9197.86 |
| 2 | 2 2 | -8437.08 | -8411.53 |
| 3 | 1 1 1 | -8437.06 | -8407.86 |
| 4 | 2 2 2 2 | -8453.25 | -8398.49 |

* Smaller absolute values indicate a better balance between fit and parsimony
