# Supplementary material for: The Neighbourhood Built Environment and Trajectories of Depression Symptom Episodes in Adults: A Latent Class Growth Analysis
Source: PLoS One. 2015 Jul 24;10(7):e0133603. doi: 10.1371/journal.pone.0133603 (PMC4514736; doi:10.1371/journal.pone.0133603)
Supplement: S3 Table — Model was weighted using Statistics Canada survey weights and incorporated for age, sex, marital status, education, income adequacy, childhood life events, chronic condition and family history of depression. (DOCX) [file pone.0133603.s004.docx]

**S3 Table. Parameter estimates for latent class growth model of major depression using 3-class solution**

| Group | Parameter | Estimate | Standard Error |
| --- | --- | --- | --- |
| 1 Low prevalence of depression symptom episode | | | |
|  | Intercept | -4.38 | 0.43 |
|  | Linear | -0.12 | 0.13 |
| 2 Moderate prevalence of depression symptom episode | | | |
|  | Intercept | -1.84 | 0.69 |
|  | Linear | -0.04 | 0.07 |
| 3 High prevalence of depression symptom episode | | | |
|  | Intercept | 0.10 | 0.46 |
|  | Linear | -0.01 | 0.13 |

Model was weighted using Statistics Canada survey weights and incorporated for age, sex, marital status, education, income adequacy, childhood life events, chronic condition and family history of depression.
