# Supplementary material for: The Neighbourhood Built Environment and Trajectories of Depression Symptom Episodes in Adults: A Latent Class Growth Analysis
Source: PLoS One. 2015 Jul 24;10(7):e0133603. doi: 10.1371/journal.pone.0133603 (PMC4514736; doi:10.1371/journal.pone.0133603)
Supplement: S4 Table — Model was weighted using Statistics Canada survey weights and incorporated for age, sex, marital status, education, income adequacy, childhood life events, chronic condition and family history of depression. (DOCX) [file pone.0133603.s005.docx]

**S4 Table. Associations between neighbourhood characteristics in 2002, 2006 and 2010 and trajectory membership.**

|  | Trajectory 1 | Trajectory 2 | Trajectory 3 |
| --- | --- | --- | --- |
|  | Low prevalence of depression symptom episodes | Moderate prevalence of depression symptom episodes | High prevalence of depression symptom episodes |
|  | n=6003 | n=949 | n=162 |
| **Neighbourhood characteristics** | RRR (95% CI) | RRR (95% CI) | RRR (95% CI) |
| **Presence of any park** | Reference |  |  |
| 2002 | 1 | 0.86 (0.63-1.18) | 0.90 (0.39-2.12) |
| 2006 | 1 | 0.83 (0.62-1.10) | 0.86 (0.41-1.84) |
| 2010 | 1 | 0.67 (0.51-0.90) | 1.19 (0.59-2.40) |
| **Presence of any health service** |  |  |  |
| 2002 | 1 | 0.67 (0.44-0.87) | 0.87 (0.36-2.09) |
| 2006 | 1 | 0.67 (0.50-0.92) | 0.87 (0.37-2.03) |
| 2010 | 1 | 0.93 (0.68-1.30) | 2.17 (1.03-4.56) |
| **Presence of any healthy food store** |  |  |  |
| 2002 | 1 | 0.69 (0.51-0.95) | 1.36 (0.64-2.86) |
| 2006 | 1 | 0.86 (0.64-1.16) | 1.11 (0.50-2.48) |
| 2010 | 1 | 1.23 (0.91-1.67) | 1.56 (0.73-3.35) |
| **Presence of any fast food restaurant** |  |  |  |
| 2002 | 1 | 0.81 (0.60-1.09) | 1.12 (0.54-2.33) |
| 2006 | 1 | 0.98 (0.73-1.32) | 1.03 (0.48-2.18) |
| 2010 | 1 | 1.19 (0.87-1.61) | 1.56 (0.78-3.23) |
| **Presence of any cultural service** |  |  |  |
| 2002 | 1 | 0.82 (0.57-1.16) | 1.54 (0.67-3.53) |
| 2006 | 1 | 1.09 (0.77-1.54) | 1.20 (0.48-3.01) |
| 2010 | 1 | 0.99 (0.69-1.43) | 2.22 (0.94-5.19) |

All models were weighted using Statistics Canada survey weights and incorporated for age, sex, marital status, education, income adequacy, childhood life events, chronic condition and family history of depression.
