# Supplementary material for: The Neighbourhood Built Environment and Trajectories of Depression Symptom Episodes in Adults: A Latent Class Growth Analysis
Source: PLoS One. 2015 Jul 24;10(7):e0133603. doi: 10.1371/journal.pone.0133603 (PMC4514736; doi:10.1371/journal.pone.0133603)
Supplement: S5 Table — All models were weighted using Statistics Canada survey weights and incorporated for age, sex, marital status, education, income adequacy, childhood life events, chronic condition and family history of depression. (DOCX) [file pone.0133603.s006.docx]

**S5 Table. Association of time-varying neighbourhood variables with the log-odd of a depression symptom episode within each trajectory class, in study participants who did not move.**

|  | Trajectory 1 | Trajectory 2 | Trajectory 3 |
| --- | --- | --- | --- |
|  | Low prevalence of depression symptom episodes | Moderate prevalence of depression symptom episodes | High prevalence of depression symptom episodes |
| **Neighbourhood characteristics** | Coefficient (95% CI) | Coefficient (95% CI) | Coefficient (95% CI) |
| **Presence of any park** | -0.7 (-2.1, 0.7) | -0.7 (-1.3, -0.2) | -0.2 (-2.1, 1.7) |
| **Presence of any healthcare service** | -1.9 (-5.5, 1.8) | -0.2 (-0.7, 0.3) | 0.6 (-0.1, 1.4) |
| **Presence of any healthy food store** | -0.2 (-0.9, 0.5) | -1.1 (-7.7, 5.6) | 0.1 (-0.6, 0.8) |
| **Presence of any fast food restaurant** | -2.0 (-5.2, 1.1) | -0.03 (-0.8, 0.7) | 0.2 (-0.5, 0.9) |
| **Presence of any cultural service** | 1.5 (-18.6, 21.6) | -0.4 (-2.1, 1.4) | 0.00 (-0.7, 0.7) |

All models were weighted using Statistics Canada survey weights and incorporated for age, sex, marital status, education, income adequacy, childhood life events, chronic condition and family history of depression.
